# Defining a set of teaching EPAs tailored to an undergraduate medical curriculum using a modified Delphi approach

**DOI:** 10.1186/s12909-024-05553-5

**Published:** 2024-05-28

**Authors:** Harm Peters, Ylva Holzhausen, Anja Czeskleba, Marwa Schumann

**Affiliations:** Dieter Scheffner Center for Medical Education und Educational Research, Dean´s Office for Study Affairs, Charité – Universitätsmedizin Berlin, Charitéplatz 1, Berlin, 10117 Germany

**Keywords:** Faculty development, Teaching EPAs, Delphi method

## Abstract

**Background:**

The concept of entrustable professional activities (EPAs) has recently been extended to operationalize professional tasks in teacher training and faculty development in health professions education. The aim of this study is to report on the process and results of defining a set of teaching EPAs (t-EPAs) tailored to the local characteristics of a particular undergraduate medical program.

**Methods:**

The undergraduate medical program at the Charité – Universitätsmedizin Berlin is competency-based, integrates thematic modules and spans 6 years. A writing team identified teaching EPAs based on the program’s study regulations and drafted content descriptions with titles, specifications and knowledge, skills and attitudes. Content validation involved a modified Delphi procedure with a systematic, iterative interaction between a panel of content experts consisting of purposively selected educators and physicians from our faculty (*n* = 11) and the writing team. The threshold for a consensus was an agreement of 80% of the participants.

**Results:**

After two Delphi rounds, a consensus was reached regarding the teaching activities to be included and their content descriptions. The response rate was 100% in both Delphi rounds. The Delphi results include the content descriptions of a total of 13 teaching EPAs, organized into the two overarching EPA domains of classroom-based (*n* = 10) and workplace-based (*n* = 3) activities. Tailoring the classroom EPAs to small group teaching and the workplace EPAs to supervising medical students led to several distinct EPAs. Another feature was the development of 2 teaching EPAs for interdisciplinary teaching.

**Conclusions:**

In systematic, Delphi-based process, we defined a set of 13 distinct teaching EPAs tailored to a specific undergraduate medical program that cover the core teaching tasks for faculty in this program. Our report on the principles of the process and the results may guide other medical schools and educators in defining and tailoring teaching EPAs according to their contexts.

**Supplementary Information:**

The online version contains supplementary material available at 10.1186/s12909-024-05553-5.

## Introduction

 Competency-based education (CBE) has evolved into a central framework in medical education globally, both for teaching students and for the training of teachers themselves [1, 2]. CBE represents a person-centered approach focusing on the competencies to be acquired by the learner [3]. To facilitate the translation of CBE into practice, the concept of entrustable professional activities (EPAs) has been introduced to provide task-centered operationalization within medical training [4]. More recently, the EPA concept has been extended to the training of teachers in health professions education. So-called teaching EPAs (t-EPAs) have been reported, for instance, for single teaching tasks specifically or as overarching EPA sets for university teachers from a Dutch academic center for health professions in general. The latter example poses limited transferability to other national or local institutional contexts [5, 6]. There is a need for sets of teaching EPAs that are better adapted to their specific context, for instance, a particular health professions program in which the EPAs will be implemented or to the educational formats to be covered [5]. Such a set could be used to more specifically guide and inform the teachers about their teaching tasks as well as for a framework for faculty development [7]. The purpose of this study is to report on the process and the results of defining a set of teaching EPAs tailored to the local characteristics of a particular undergraduate medical program.

CBE-based training of teachers has become a central component of faculty development in higher education, including medicine. Several frameworks have been developed to conceptualize CBE using a person-centered approach [1, 2], i.e., defining the competencies and roles that an individual teacher should acquire and demonstrate for teaching. Central to CBE is the notion that teachers should display competencies that equip them to go beyond being content experts to fulfil roles such as learning facilitators and coaches [8, 9]. While CBE specifies the competencies needed to teach, the approach is not easily translated into teaching practice; for instance, CBE does not specify which competencies are needed in which particular teaching format and which competency is needed at a particular phase of the teaching sequence in a particular course [5].

The concept of EPAs represents a task-based approach that aims to facilitate the transfer of CBE into practice, i.e., to link the competencies of an individual with the tasks to be done in workplace practice [10]. EPAs include the real-life tasks and responsibilities of a profession that are “independently executable within a time frame, observable and measurable in the process and outcome” [10]. The concept of EPA was initially introduced in postgraduate medical training, where clinical tasks are entrusted to a trainee once sufficient competence is reached to allow for more autonomous practice [4]. In the last decade, the EPA concept has been extended to undergraduate medical education as well as to veterinary medicine, dentistry, and nursing training [11–13].

More recently, the EPA concept has been proposed and applied to teaching tasks in the health professions, using this concept to operationalize teaching activities in a more tangible manner while to some extent bracketing a discussion about entrustment and supervision [6, 14]. Teaching EPAs have been reported for specific single teaching formats such as bedside teaching and more broadly for small group facilitation in an elaborate manner [7, 15, 16]. In a more condensed approach, van Bruggen et al. defined a set of nine teaching tasks that most university health professions teachers undertake [5]. They identified these tasks by using a two-round Delphi consensus procedure based on the Dutch National Teacher Qualification Framework and by seeking expert consultation from a Dutch academic center [5]. In a subsequent international survey and focus group discussion on the resulting nine EPA descriptions, there was far less consensus about which teaching tasks should be included and their content specification, and a need for better adaptation to the specific local contexts was expressed. As such, the factors identified as needed taken to be into account ranged from different definitions and specific aspects of teaching tasks; the type of program in health professions education, e.g. medicine or nursing, or both; the use of different wording in particular national and local contexts; and the relevance of organizational, educational and cultural differences in local environments. Sets of teaching EPAs adapted to the local contexts have been recommended to serve as a next step in the development of this field. These sets of teaching EPAs may allow for the collection of more specific and tangible information on the performance of teaching tasks and for the preparation of teachers; they may also serve as a framework for faculty development in a given institutional context [5].

The aim of this study is to report on the principles of the process and the results of defining a set of teaching EPAs tailored to the local characteristics of a particular undergraduate medical program. For content validation, we employed a modified Delphi consensus procedure with a group of local educational experts. The goal was to identify a set of core teaching EPAs for most of the faculty teachers in this program and to elaborate the EPAs according to a modified 5 category description including ‘title’, ‘specification and limitation’; ‘Knowledge, Skills and Attitudes’ (KSA) and teacher competencies domains [17].

## Materials and methods

### Setting

This study was conducted at the Charité – Universitätsmedizin Berlin (Charité), a large European medical university. Its undergraduate medical program (Modular Curriculum of Medicine, MCM) covers a total of six years and represents a fully integrated, competency-based program with 40 thematic modules and a final clerkship year [18]. Teaching formats include a blend of lectures, seminars, laboratory practicals, skills training courses, bedside teaching, problem-based learning (PBL) and communication training (Communication, Interaction and Teamwork, CIT) supported by a student-led peer-assisted learning program [19]. The program was designed and implemented between 2010 and 2016, replacing the previous traditional, discipline-based undergraduate medical curriculum [18].

The faculty comprises 2500 members with diverse professional backgrounds in clinical and basic science disciplines, and there are mandatory teaching qualifications. Notably, all faculty members at the Charité are involved in teaching, but with varying degrees of involvement in assessment and curriculum development. While teaching responsibilities are universally shared, fewer faculty members are involved in assessment activities, and an even smaller subset is involved in curriculum development. To address this dynamic, the study began by defining the roles that are relevant to the majority of faculty. Another reason is that although our faculty are familiar with clinical EPAs, they are not yet familiar with teaching EPAs. To address this unfamiliarity, we have adopted a phased change management approach. Initially, we have focused on defining EPA-aligned teaching activities to build a foundational understanding of EPAs and lay the groundwork for their integration into other academic roles such as assessment and curriculum development.

The study protocol was approved by the Data Protection Office and the Ethics Committee of the Charité (No. 0554/17/ST3). Informed consent was required for participation.

### Study design

**Fig. 1:**
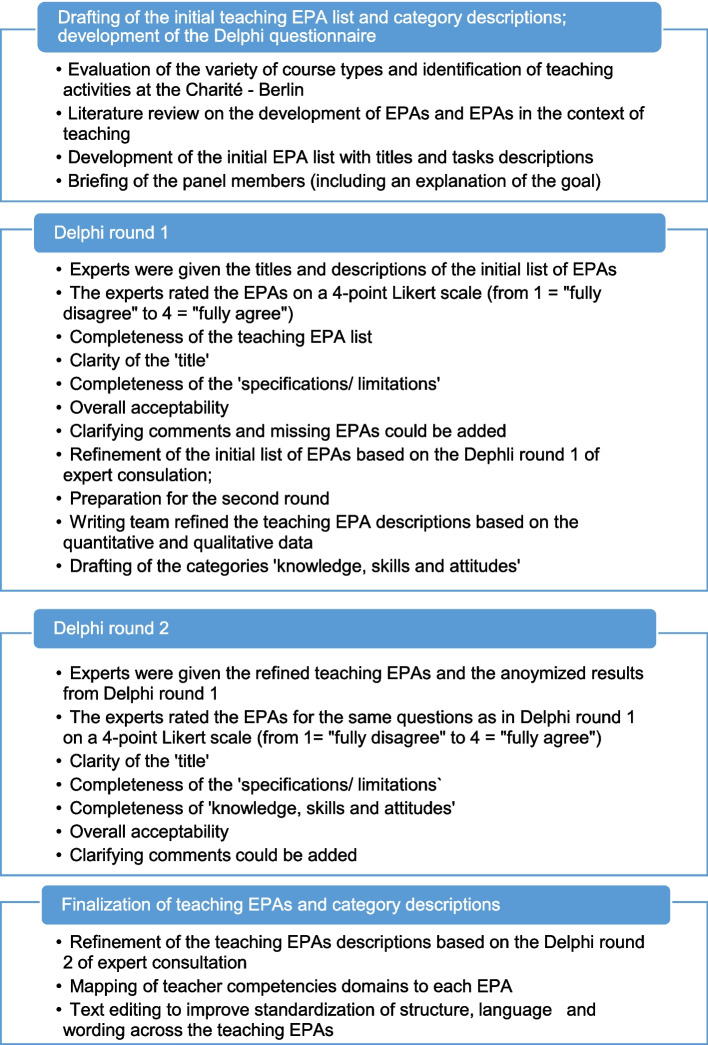
Overview of the step-by-step, modified Delphi process for developing teaching EPAs

We employed a modified Delphi technique, which is an established method for conducting anonymous and non-hierarchical content validation in EPA development [17, 20]. The aim was to define a full set of teaching EPAs representing authentic teaching activities in the undergraduate medical curriculum at the Charité. The breath and scope of each teaching EPA was elaborated iteratively according to EPA writing guidelines [17]. The content of the teaching EPAs was elaborated and validated through a step-by-step interaction between a writing team and a panel of content experts [21, 22]. Figure 1 provides an overview of the Delphi study procedure, i.e., the main steps and tasks involved.

### Panel selection and writing team

The Delphi panel consisted of purposively selected educationalists and physicians from the Charité faculty. The selection was based on the individuals providing didactic faculty development courses, playing a key role in the central curriculum development process or management of specific teaching formats and having a familiarity with Charité teaching formats and the EPA concept. Each panellist gave written consent to participate in the study. The writing team consisted of the authors of this manuscript (HP, AC, YH and MS), with combined expertise in the MCM curriculum and didactic faculty development at the Charité and in the elaboration and validation of EPAs.

### Guiding principles in the process of teaching EPA content definition

The teaching EPAs were developed in line with recommendations for the definition of EPAs representing independently executable tasks that are observable, measurable and confined to qualified personnel [23]. The breadth and scope of each teaching EPA was elaborated according to the categories of ‘title’, ‘specification and limitation’; ‘knowledge, skills and attitudes’ (KSA) and teacher competencies domains, based on the 8 roles of the medical teacher as a competency framework [2, 17, 24]. The set of teaching EPAs should constituted the range of professional teaching activities expected of most teachers in our undergraduate medical program following the explicit chronological order, i.e., listing the tasks before, during and after the teaching activity. The description is built on common overarching, observable task-based educational frameworks. For the classroom-based teaching EPAs, we used the Teaching Quality Framework which is a questionnaire-based and validated tool that focuses on the assessment of didactic competence based on the three domains of the cognitive aspect of knowledge transfer and understanding, the motivational aspect of learning and the social aspect of managing interaction within the learning group [25, 26]. For the workplace-based teaching EPAs, we chose a clinical EPA framework, i.e., core EPAs for entry into residency according to the clinical practice in our context [27]. Similar to von der Bruggen et al., we did not address the questions of assessment, entrustment and increasing levels of autonomy [5].

### Identification of teaching EPAs and drafting of the content descriptions

The writing team identified potential teaching EPAs in the MCM program and iteratively elaborated their respective content. The identification of potential teaching EPAs was based on a systematic search of the national licensing law for undergraduate medical education, the MCM study regulations, the MCM online platform and student learning materials. The identification of the teaching EPAs was finally based on the teaching formats defined in the MCM study regulations. The elaboration of the content of the each teaching EPA was developed based on information materials (handouts, teaching and learning guides) on the respective teaching format provided to students or in faculty development activities for teachers.

### Questionnaire design and establishment of consensus

The writing team drafted a questionnaire for the modified Delphi study. The questionnaire included the proposed complete list of titles of teaching EPAs and content descriptions of each EPA category. The panelists rated the completeness of the list of teaching EPAs; the clarity of the titles; the completeness of the task descriptions; the completeness of the corresponding knowledge, skills and attitudes; and the overall description of the teaching EPAs. In each Delphi round, the panellists individually rated perspective statements on the EPA aspects on a four-point Likert scale (1 = fully disagree, 2 = partly disagree, 3 = partly agree and 4 = fully agree) and provided narrative comments on potential improvements. The modified Delphi study questionnaire was designed and distributed using Microsoft Word.

The predetermined level of consensus is ≥ 80%, meaning that at least 80% of respondents rate the respective perspective statements as fully agree or partly agree [20, 28].

### Panel member invitation and briefing

In January 2018, a meeting was held with the panelists to introduce them to the aim, scope and process of the modified Delphi study. The introduction was followed by an interactive discussion to allow for clarification. The panellists were also briefed on the concept of EPAs. As we have a well-established EPA framework for the final clerkship year in medicine, all the panellists were already familiar with EPAs, which complemented their expertise in medical education.

#### Round 1

The first Delphi round started in January 2018, when the panelists were sent the first draft of the teaching EPA title and the description of the task specification as prepared by the writing team. The panelists evaluated the teaching EPA aspects and provided written comments. All panelist responses were received by April 2018. The writing team analyzed and summarized both the quantitative ratings and the narrative comments. The content and elaboration of the core EPAs were adjusted based on an iterative process in the writing team. In addition, the writing team drafted a text for the KSA category for all teaching EPAs.

#### Round 2

The second Delphi round started in May 2019. The panelists received the anonymized results of the first Delphi round, the refined teaching EPA titles and task specifications and the draft of the KSA descriptions for all t-EPAs. All panelist responses were received by August 2019. The writing team analyzed and summarized both the quantitative ratings and the narrative comments. The Delphi study process was stopped after the panelists’ ratings and qualitative comments were received.

### Finalization

The writing team made final changes to the content descriptions of the teaching EPAs based on the panel members’ ratings and comments from Delphi round 2. The teacher competencies domains were mapped to individual teaching EPAs by the writing team. Furthermore, special attention was paid to harmonizing the structure, language, and wording of the EPA descriptions. The EPAs were also arranged according to the overarching EPA domains and classroom-based and workplace-based learning.

### Data analysis

Descriptive statistics were calculated using IBM SPSS Statistics™ (version 26, 2019). The panellists’ ratings are reported as the mean and the calculated consensus for each Delphi round.

## Results

### Panel members and Delphi round response rates

In Delphi rounds 1 and 2, the response rate was 100% (11 out of the 11 invited panellists). Seven panelists were physicians (all with additional didactic qualifications, in four cases a Master of Medical Education (MME) degree) and four had a degree in psychology, education or nursing. There were 7 male and 4 female panellists with teaching experience ranging from 3 to 22 years, with a mean of 11.5 years. Five of the panellists had experience in curriculum development, and 10 panelists had experience in planning individual teaching courses.

### Resulting EPAs

A total of 13 teaching EPAs were identified by the writing team based on the MCM study regulations; these teaching EPAs which were further subdivided into ten classroom-based and three workplace-based teaching EPAs (Table 1). This set of teaching EPAs was confirmed by the panellists, and no additional teaching EPAs were suggested.

**Table 1:** Results of the panellists’ ratings of the content descriptions of the 13 teaching EPAs in Delphi round 1 (R 1) and round 2 (R 2). Shown is the relative proportion of panellist agreeing on the perspective statements and the mean rating (in parentheses) on a 4-point Likert scale (1 = fully disagree to 4 = fully agree). In Delphi round 2, the category ‘’Knowledge, Skills and Attitudes’ (KSA) was included

<	EPA title	Consensus (%) ‘Title’		Consensus (%) ‘Specification’		Consensus (%) ‘KSA’		Consensus (%) Overall acceptance	
		R 1	R 2	R 1	R 2	R 1	R2	R 1	R 2
**1.**	**Class room-based teaching**								
1.1.	Give a lecture	100% (3.8)	100% (4.0)	80% (3.2)	100% (3.8)		100% (3.9)	40% (2.4)	100% (3.9)
1.2.	Teach a seminar	100% (3.9)	100% (4.0)	90% (3.2)	90% (3.4)		100% (3.6)	10% (2.1)	90% (3.5)
1.3.	Teach an interdisciplinary seminar	100% (3.8)	100% (4.0)	89% (3.2)	90% (3.4)		100% (3.4)	30% (2.6)	90% (3.4)
1.4.	Teach a laboratory course	100% (4.0)	100% (3.8)	88% (3.3)	100% (3.5)		100% (3.5)	22% (2.2)	100% (3.7)
1.5.	Teach a bedside teaching course	100% (4.0)	100% (3.9)	90% (3.6)	100% (3.7)		100% (3.8)	60% (2.6)	100% (3.9)
1.6	Teach a communication interaction teamwork course	100% (3.9)	100% (4.0)	60% (2.8)	82% (3.5)		82% (3.4)	20% (2.2)	91% (3.6)
1.7.	Facilitate a problem-based learning group	100% (3.9)	100% (4.0)	90% (3.6)	100% (3.6)		100% (3.5)	78% (2.8)	100% (3.6)
1.8.	Teach a clinical skills course	100% (4.0)	100% (3.9)	100% (3.7)	100% (3.7)		100% (3.7)	75% (3.1)	100% (3.9)
1.9.	Teach an emergency simulation course	88% (3.9)	100% (4.0)	100% (3.7)	100% (3.6)		100% (3.8)	33% (2.3)	100% (3.9)
1.10	Teach an interdisciplinary emergency simulation course	88% (3.6)	100% (4.0)	100% (3.3)	100% (3.6)		100% (3.6)	29% (2.3)	100% (3.8)
**2.**	**Workplace-based teaching**								
2.1.	Supervise a medical student in short-block clinical placements	100% (4.0)	100% (3.9)	100% (3.9)	100% (3.7)		100% (3.9)	86% (2.9)	100% (3.9)
2.2.	Supervise a medical student in early clerkship placements	100% (4.0)	100% (4.0)	100% (4.0)	100% (3.8)		100% (4.0)	71% (2.7)	100% (4.0)
2.3	Supervise a medical student in final clerkship year placements	100% (4.0)	100% (4.0)	100% (3.7)	100% (3.8)		100% (4.0)	57% (2.6)	100% (4.0)

Whether classroom-based or workplace-based, the defined teaching EPAs were very diverse in terms of group size, learner focus, interactivity (among learners or between learners and teachers), delivery of practical or theoretical content and even the use of simulated patients (Table 1). For teaching formats with preexisting standardized processes (e.g., PBL, CIT, bedside teaching), the described processes and requirements were accounted for and integrated into the teaching EPAs. An example is given in Table 2: ‘Facilitate a problem-based learning group.

**Table 2:**
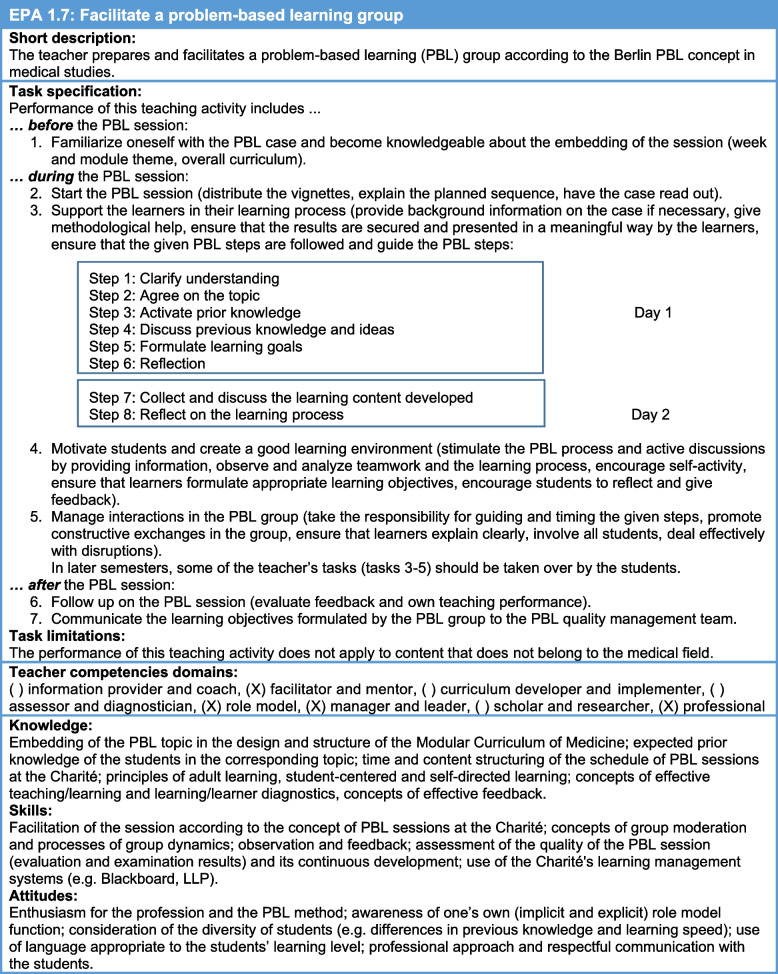
Classroom-based teaching EPA 7: Facilitate a problem-based learning group

The common feature of the workplace-based teaching EPAs was that students learn on the ward/in practice, outside the normal university context, thereby immersing themselves in their future working environment. An example of a workplace-based teaching EPA is given in Table 3.: ‘Supervise a medical student in final clerkship year placements. There are three separate workplace-based teaching EPAs as the embedded clinical EPAs and the respective levels of supervision for the medical students differ substantially between the three types of clerkships (see Appendix II).

**Table 3.** Workplace based teaching EPA 3: Supervise a medical student in the final clerkship year placements

**EPA 2.3: Supervise a medical student in the final clerkship year placements**
**Short description:** The teacher instructs and supervises a medical student in the final clerkship year placement and prepares him/ her for the start of postgraduate medical training.
**Task specification:** Performance of this activity includes ... ***… before*** the start of the final clerkship year rotation: 1. Welcome the student and introduce him /her to the ward or outpatient clinic (health care team, overview of the range of complaints, diseases, procedures, therapies, learning opportunities). 2. Communicate what the medical student are allowed to do by him- / herself (with supervisor´s follow-up check) or together with a physician or are not allowed to perform (considering previous clinical and practical experience and/or the EPAs already performed). 3. Describe the expectations of what should be achieved in the corresponding rotation of the final clerkship year (tasks and level of autonomy/supervision) and show the gaps compared to the intended outcomes (educational objectives of the Charité) at the end of the final clerkship year. ***… during*** the final clerkship year rotation: 4. Motivate the student and create a supportive learning environment (arouse interest, clarify relevance, encourage, formulate appropriate task requirements, encourage self-activity). 5. Assign tasks of increasing difficulty to the student. 6. Guide the student through unfamiliar activities (instruct, co-perform, observe and provide feedback). 7. Supervise tasks carried out autonomously by the student (check work results). 8. Provide the final clerkship student with a place to work and study. 9. Actively integrate the student into the team (assigns roles and tasks, manages communication and time, deals effectively with disruptions). ***… after*** the final clerkship year rotation: 10. Discuss the outcomes of the corresponding/respective final clerkship year rotation (tasks and level of autonomy/ supervision) and addresses any gaps in relation to the start of postgraduate medical training. 11. Reflect on one’s own performance (e.g., teaching style, feedback, time management). **Task limitations:** The performance of this teaching activity does not apply to content that does not belong to one’s own subject.
**Teacher competencies domains:** ( ) information provider and coach, (X) facilitator and mentor, ( ) curriculum developer and implementer, (X) assessor and diagnostician, (X) role model, ( ) manager and leader, ( ) scholar and researcher, (X) professional
**Knowledge:** Content knowledge in the relevant area (range of complaints, diseases, procedures, therapies); expected prior knowledge and level of clinical skills of a final clerkship year student; principles of effective teaching, learning and assessment in the clinical workplace; concept of clinical education and 1:1 teaching; structuring of learning and participation of final clerkship year students in real patient care settings; use of Entrustable Professional Activities (EPAs) in a clinical context; objectives for the final clerkship year (range of tasks to be performed and level of autonomy/ supervision achieved); steps and teachings of clinical reasoning in differential diagnosis and treatment decisions; principles of effective feedback; the importance of being implicit and explicit role modelling. **Skills:** Introduction of the clerkship year student to the workplace and the team; explicit specification of the range of tasks and the level of autonomy/ supervision; identification of learning opportunities during the final clerkship year in the relevant specialty; setting of objectives to be achieved by the end of the practical year; teaching of practical skills; observation and feedback; support of the student in their professional development; final discussion with a reflection on what has been achieved and what is being aspired to in the future. **Attitudes:** Enthusiasm for the subject and profession; awareness of being an (implicit and explicit) role model; respectful treatment of patients and colleagues in the (interprofessional) health care team; compliance with hygiene regulations; respect for and emphasis on privacy and the confidentiality of patient information; consideration of the diversity of students (e.g. differences in prior knowledge and speed of learning); professional interaction and respectful communication with the students; roles as a teacher, coach and mentor for the professional development of the final clerkship year students.

### Content description and validation of the teaching EPAs

Table 1 shows the results of the panelists’ ratings of the content descriptions of the 13 teaching EPAs for undergraduate medical education at the Charité. In Delphi round 1, the draft of the categories of ‘title’ and ‘specification/limitation’ resulted in a high agreement for 12 of the 13 teaching EPAs, while only 1 teaching EPA showed an overall acceptance of more than 80%. In Delphi round 2, the descriptions of ‘title’, ‘specification/limitation’ and ‘knowledge, skills and attitudes’ for all teaching EPAs received the required agreement for all teaching EPAs to reach consensus. In addition, the agreement for overall acceptance was greater than 80% for all teaching EPAs, indicating that sufficient consensus was reached among the panelists. Based on the panelists’ ratings and narrative comments, the content descriptions of the teaching EPAs were refined by the writing team as part of the step to finalize the teaching EPA descriptions. In the second round, additional descriptions of the requirements were provided with the description of Knowledge, Skills and Attitudes (KSA). This was particularly important for PBL and CIT, for which specific requirements and training at the Charité are mandatory.

## Discussion

The concept of EPAs has been extended to operationalizing tasks in relation to teaching and faculty development in the field of health professions education. The aim of this approach is to complement abstract CBE teaching frameworks into practice by using tangible task-based operationalizations. In this study, we defined a set of 13 teaching EPAs specifically tailored to the teaching formats of a CBE-based, undergraduate medical program at our institution, thereby adding to the previous reports on this new and developing area of educational research. Using a systematic process that involved a modified Delphi approach with a writing team and a panel of content experts, we described and validated the content of these teaching EPAs and aligned them to specific teaching formats of the undergraduate medical program in our institution. In the following sections, we discuss the findings of this study in the context of the existing literature.

In this study, we utilized a number of principles to define the descriptions of the teaching EPAs. These included adherence to recommendations for the definition of EPAs, i.e. tasks representing independently executable tasks that are observable, measurable and confined to qualified personnel [23] and that were elaborated according to the categories of ‘title’, ‘specification and limitation’; ‘knowledge, skills and attitudes’ (KSA) and teacher competencies domains based on the 8 roles of the medical teacher as a competency framework [2, 17, 24]. Further principles were that the tasks were described in chronological order, i.e., listing the tasks before, during and after the teaching activity. In addition, we used locally established educational frameworks to elaborate the teaching EPAs, i.e. for the classroom-based teaching EPAs the Teaching Quality Framework with the three domains of the cognitive aspect of knowledge transfer and understanding, the motivational aspect of learning and the social aspect of managing interaction within the learning group [25, 26] and for workplace-based teaching EPAs a clinical EPA framework, i.e., core EPAs for entry into residency according to the clinical practice in our context [27]. Furthermore, we chose to use a modified Delphi procedure as an established method for defining the content of the teaching EPAs. In particular, compared with other methods of EPA definition, such as expert meetings, the Delphi method allows for content validation in a non-hierarchical, anonymized manner. In addition, when used in conjunction with digital technology, the method is a convenient, cost-effective tool for interacting with content experts in a timely manner. A special feature of our Delphi study that should be emphasized is that the panel of content experts consisted of an interdisciplinary group of both educational experts and teaching faculty from our institution. Many of the participating physicians had a degree in medical education and thus a solid grounding in educational theory. Another special feature of our study is that it took only two Delphi rounds to reach a consensus on the set of teaching EPAs. The key to this efficiency was establishing a writing team with expertise in both the definition of EPAs and the content of the envisaged teaching EPAs.

The results of this study are content descriptions of a set of 13 teaching EPAs tailored to our institution’s competency-based undergraduate medical program. These teaching EPAs range from traditional academic formats such as “give a lecture” to new formats such as “teach a communication interaction teamwork course”. Ten teaching EPAs fall into the overarching domain of classroom-based activities, and 3 fall into the domain of workplace-based teaching activities. To date, only few cases have been reported in the literature where the concept of EPAs has been used for teaching tasks, ranging from specific teaching tasks such as bedside teaching [15], small group facilitation [16] or program direction [29], to more comprehensive approaches such as the definition of a set of teaching tasks for university teachers in health professions education [5] or the EPA-based curriculum framework of the Master of Health Professional Education at Michigan University (UM-MHPE) [14].

Our teaching EPAs were developed in parallel and independently of the nine generic teaching EPAs for university teachers by van Bruggen et al. [5]. The two sets align in principle with each other. While some teaching EPAs are very similar, such as “give a lecture” and “teach a bedside teaching course”, others were more differentiated and resulted in several distinct EPAs. For example, we defined separate teaching EPAs for different formats of small group teaching, such as “teach a communication interaction teamwork course” and “facilitate a problem-based learning group”. For the generic “supervise (clinical) interns” activity, we defined separate teaching EPAs according to the specific placement of the medical student at different stages of the undergraduate program, such as for early clerkships (semesters 5 to 10), short-block placements (semester 10) and the final clerkship year. Furthermore, we included a distinct EPA for teaching a seminar compared to that of van Bruggen et al., who included teaching a seminar in the lecturing EPA [5]. In addition, as an indicator of differentiation, we included distinct teaching EPAs for when seminars and emergency simulations are taught interdisciplinary by faculty from different disciplines.

When teaching EPAs are tailored to the context of a particular undergraduate medical program, they become more granular and more specific. In our view, they provide a more tangible operationalization of the teaching tasks. In our context, this is important, as we have a large number of new clinical teachers each semester. On an individual level, these teachers in our context generally do only a few hours of teaching per semester and have little time to accustom themselves to their teaching tasks. Tailoring of teaching EPAs allows us to include locally established specific aspects of teaching tasks, for instance, those underpinning educational theory or organizational aspects. Our teaching EPAs are specific to teaching in undergraduate medical education and not to other health professions programs. In addition, we have not included EPAs for mentoring, tutoring, course design or assessment tasks, as these activities are carried out by a small group of our teaching faculty.

Beyond defining a set of teaching EPAs tailored to the teaching formats of a specific undergraduate medical program, this study has several implications and perspectives. First, this study may serve as an example and guide for other faculties on how to tailor and define a set of teaching EPAs to the local setting of a given educational program using a consensus method such as a modified Delphi approach. Second, the resulting teaching EPAs can be used by other faculties in their undergraduate medical programs. This applies in particular to the national context of Germany, where many of the teaching formats in undergraduate medical education are similar, as well as to an international context with further adaptation of the teaching EPAS to their local contexts. Thirdly, the teaching EPA set can be used as an outcome-based framework and curriculum guide for a structured faculty development program at our institution.

Our approach to defining teaching EPAs is in line with the EPA framework in that it is based on the task-centered approach in the operationalization of teacher responsibilities (instead of competencies a teacher should pose). The teaching EPAs represent real life tasks and responsibilities of a profession that are “independently executable within a time frame, observable and measurable in the process and outcome” [10]. Our approach is not consistent with the EPA framework in terms of assessment and entrustment decisions with different levels of supervision. A similar approach has previously used to define an overarching set of EPA for university teachers [5], a set of EPAs representing the core expectation for PhDs in health professions education [30]; and EPAs for the training of translational scientists [28].

We acknowledge that teacher entrustability in medical education means navigating in a complex landscape [31]. However, using the EPA framework primarily to operationalize teaching responsibilities in a task-based manner has merit and can advance the field. In this sense, teaching EPAs can provide a basis for guiding faculty in their personal development and career choices, describing the breadth and depth of activities expected to enhance their understanding of their professional roles [30]. As health educators are pre-qualified health professionals who are allowed to work independently under their licence, teaching EPAs are used to shape faculty development based on professional teaching activities, rather as a formal assessment tool [5, 32]. EPAs can also be useful in improving the recognition of teaching performance, and in designing or refining systems for professional development, evaluation and recognition of teaching, contributing to ‘competence’ and ‘career’ as two important areas for improving the professional development of HPE faculty [5]. Finally, the 13 teaching EPAs defined in this study can serve as a comprehensive framework for future educational research, such as implementation research analyzing the uptake and refinement in practice by teachers and at the institutional level. Research can address requirements or instruments for the evaluation and assessment of these teaching EPAs. Further and new teaching tasks may be added to the current set, for example, for teaching formats involving online learning or possibly related to leadership functions to reflect the multiple tasks of teachers in an academic setting.

The study was conducted just prior before the COVID-19 pandemic. Its onset disrupted our faculty development initiatives. We had to redirect our resources efforts to Emergency Remote Teaching (ERT). The focus shifted to online teaching methods, delaying the implementation of the defined teaching EPAs at our institution. Currently, we are cworking on the development and implementation of an e-portfolio for teachers based on the set of EPAs we have developed.

We acknowledge the limitations of this study. First, it was conducted in a single medical university. The transferability to other contexts may be limited. The number of panellists was small, which may have led to selection bias. The EPAs were defined for face-to-face teaching and cannot be transferred directly when a teaching format such as “give a lecture” is delivered in an online setting. The set of teaching EPAs is not complete, as we focused on teaching formats in which the majority of the teachers at our institution would teach, and therefore does not capture the full range of professional teaching activities in academic teaching, e.g. assessment or curriculum development.

## Conclusions

We report on the principle to the define a set of distinct teaching EPAs that are tailored to the specifics of an undergraduate medical program of one local context. The resulting EPAs are in more granular and specific content descriptions than the reported overarching teaching EPAS for university teachers [5]. They cover the core teaching tasks for faculty in this program. Each educational setting has its own characteristics, including unique curricular frameworks, teaching methods and organizational aspects. Tailoring teaching EPAs to the local context enhances their applicability and relevance to the particular educational setting and the teaching faculty. They can enable targeted faculty development strategies and interventions. This report may stimulate and guide other medical schools and educators in the principles of defining and tailoring teaching EPAs according to their contexts.

### Supplementary Information

Supplementary Material 1.

Supplementary Material 2.

## Data Availability

The data set used and analyzed during this study is not publicly available due to ethical and regulatory restrictions but is available from the corresponding author upon reasonable request.
