# Supplementary material for: Defining a set of teaching EPAs tailored to an undergraduate medical curriculum using a modified Delphi approach
Source: BMC Med Educ. 2024 May 28;24:588. doi: 10.1186/s12909-024-05553-5 (PMC11134953; doi:10.1186/s12909-024-05553-5)
Supplement: Supplementary file 1 — Supplementary Material 1. [file 12909_2024_5553_MOESM1_ESM.docx]

**Appendix I - “Defining a set of teaching EPAs tailored to**

**an undergraduate medical curriculum using a modified Delphi approach.”**

**Set of 13 teaching EPAs for the undergraduate medical curriculum**

**at the Charité - Universitätsmedizin Berlin**

| 1 | **Classroom-based teaching** |
| --- | --- |
| 1.1. | Give a lecture |
| 1.2. | Teach a seminar |
| 1.3. | Teach an interdisciplinary seminar |
| 1.4. | Teach a laboratory course |
| 1.5. | Teach a bedside teaching course |
| 1.6. | Teach a communication interaction teamwork course |
| 1.7. | Facilitate a problem-based learning group |
| 1.8. | Teach a clinical skills course |
| 1.9. | Teach an emergency simulation course |
| 1.10. | Teach an interdisciplinary emergency simulation course |
| 2 | **Workplace-based teaching** |
| 2.1. | Supervise medical a student in early clerkship placements |
| 2.2 | Supervise medical a student in short-block clinical placements |
| 2.3 | Supervise medical a student in final clerkship year placements |

| **EPA 1.1: Give a lecture** |
| --- |
| **Short description:**  The teacher prepares, gives and follows up on a lecture in his/ her discipline for medical students in terms of content and didactics. |
| **Task specification:**  Performance of this teaching activity includes ...  ***… before*** the lecture:   1. Become informed about the content, learning objectives to be taught, and context of the lecture (in the week and module theme and overall curriculum) and check that they are up to date. 2. Prepare for the sequence of the lecture (content arrangement, time structure, teaching methods and materials, organizational matters).   ***… during*** the lecture:   1. Start the lecture (introduction, learning objectives, embedding in the module and in the curriculum, and planned sequence). 2. Provide the students with the learning content in an appropriate way and support them in their learning process (provide a clear and structured presentation, provide comprehensible explanations, summarize and repeat, activate previous knowledge and link it with new learning content, make connections, check understanding, answer students' questions when necessary, give feedback to stimulate learning, use didactic aids appropriately, use appropriate language) 3. Motivate students and create a good learning environment (arouse interest, avoid monotony, encourage self-activity, clarify relevance, encourage, formulate appropriate task requirements). 4. Manage interactions in the student group (manage communication and use of time, deal effectively with disruptions). 5. Close the lecture (summarize, poss. mention further learning resources, allow time for online evaluation).   ***… after*** the lecture:   1. Follow up on the lecture (evaluate feedback, revise the lecture materials [e.g. presentation slides], reflect on one’s own priority setting and teaching performance).   **Tasks limitations:**  The performance of this teaching activity does not apply to content that does not belong to the teacher´s subject area. |
| **Teacher competencies domains:**  (X) information provider and coach, ( ) facilitator and mentor, ( ) curriculum developer and implementer, ( ) assessor and diagnostician, (X) role model, ( ) manager and leader, ( ) scholar and researcher, (X) professional. |
| **Knowledge:**  Content knowledge of the lecture topic; embedding of the topic in the sequence and structure of the Modular Curriculum of Medicine; expected prior knowledge of the students in the corresponding topic; structuring of lectures in terms of time and content; basic scientific concepts of the learning process (and for forgetting); principles of adult learning, learner focus and self-directed learning; importance and dimensions of learning objectives; alignment of teaching, learning and assessment based on learning objectives; concepts of effective teaching/learning diagnostics.  **Skills:**  Design of the lecture according to the concept of teaching quality (main dimensions: transfer of knowledge and support of understanding, motivation and creation of a supportive learning environment; management of interaction in the learning group); learning/learner diagnostics at the beginning and in the course of the lecture; demonstration of relevance for the medical profession and adaptation of the lecture accordingly; assessment of the quality of the lecture (evaluation and examination results) and its continuous development; use of the Charité's learning management system (e.g. Blackboard, LLP).  **Attitudes:**  Enthusiasm for the subject and profession; awareness of one’s own (implicit and explicit) role model; consideration of the diversity of students (e.g. differences in previous knowledge and learning speed); use of language appropriate to the students’ learning stage; professional approach and respectful communication with the students. |

| **EPA 1.2: Teach a seminar** |
| --- |
| **Short description:**  The teacher prepares, teaches and follows up a seminar in his/ her discipline for medical students in terms of content and didactics. |
| **Task specification:**  Performance of this teaching activity includes ...  ***… before*** the seminar:   1. Become informed about the content, learning objectives to be taught, and context of the seminar (in the week and module theme and overall curriculum) and check that they are up to date. 2. Prepare for the sequence of the seminar (content, time structure, teaching methods and materials, organizational matters).   ***… during*** the seminar:   1. Start the seminar (introduction, learning objectives, embedding in the module and in the curriculum, and planned sequence). 2. Provide the students with the course content in an appropriate way and support them in their learning process (provide a clear and structured presentation, provide comprehensible explanations, summarize and repeat, make links, check understanding, give feedback to stimulate learning, use didactic aids in a targeted way, use appropriate language). 3. Motivate students and create a good learning environment (arouse interest, avoid monotony, encourage self-activity, clarify relevance, encourage, formulate appropriate tasks). 4. Manage interactions in the student group (manage communication and time, give individualized feedback, deal efficiently with disruptions). 5. Close the seminar (summarize, poss. mention topics for further study, elicit feedback from students, allow time for online evaluation, and fill in attendance cards).   ***… after* the seminar:**   1. Follow up on the seminar (evaluate feedback, revise the materials [e.g. presentation slides], reflect on one’s own priority setting and teaching performance).   **Task limitations:**  The performance of this teaching activity does not apply to content that does not belong to one’s own subject area. |
| **Teacher competencies:**   - (X) information provider and coach, (X) facilitator and mentor, ( ) curriculum developer and implementer, ( ) assessor and diagnostician, (X) role model, ( ) manager and leader, ( ) scholar and researcher, (X) professional. |
| **Knowledge:**  Content knowledge in the seminar topic; embedding of the topic in the sequence and structure of the Modular Curriculum of Medicine; expected prior knowledge of the students and their expectations in the corresponding topic; structuring of the seminar in terms of time and content; specific potentials of the seminar as a teaching format; basic scientific concepts of the learning process (and for forgetting); principles of adult learning, student-centered and self-directed learning; importance and dimensions of learning objectives; alignment of teaching, learning and assessment based on learning objectives; concepts of effective teaching/learning and learning/learner diagnostics.  **Skills:**  Design of the seminar according to the concept of teaching competence (main dimensions: transfer of knowledge and support of understanding, motivation and creation of a supportive learning environment; management of group interaction in the learning group); learning/learner diagnostics at the beginning and during the seminar; demonstration of relevance to the medical profession and adaptation of the teaching design accordingly; assessment of the quality of the seminar (evaluation and examination results) and its continuous development; use of the Charité’ s learning management system (e.g. Blackboard, LLP).  **Attitudes:**  Enthusiasm for the subject and profession; awareness of one’s own (implicit and explicit) role model function; consideration of the diversity of students (e.g. differences in previous knowledge and learning speed); use of a language appropriate to the students’ level; professional approach and respectful communication with the students. |

| **EPA 1.3: Teach an interdisciplinary seminar** |
| --- |
| **Short description:**  The teacher prepares, teaches and follows up an interdisciplinary seminar in his/ her discipline for medical students in terms of content and didactics. |
| **Task specification:**  Performance of this teaching activity includes ...  ***… before*** the interdisciplinary seminar:   1. Become informed about the content, learning objectives to be taught, learning objectives of the colleague and embedding of the interdisciplinary seminar (in the week and module theme and the overall curriculum) and check that they are up to date. 2. Prepare for the sequence of the seminar in collaboration with the colleague (content arrangement, agreement on content and learning objectives with the colleague from other disciplines, time structure, teaching methods and materials, organizational matters) and coordinate the content with him/ her (including time and role allocation).   ***… during*** the interdisciplinary seminar:   1. Start the interdisciplinary seminar (introduction, learning objectives, embedding in the module and in the curriculum, planned sequence, highlighting the importance of the interdisciplinary setting). 2. Teach the content in an appropriate way to the students and support them in their learning process (provide a clear and structured presentation, provide comprehensible explanations, summarize and repeat, make links, check understanding, give feedback to stimulate learning, use didactic aids in a targeted way) 3. Motivate the colleagues and create a good learning environment (arouse interest, avoid monotony, encourage self-activity, clarify relevance, encourage, formulate appropriate tasks). 4. Manage interactions in the student group (manage communication, manage time, manage conflict effectively). 5. Organize the transition(s) between the 2 parts of the seminar together with the other colleagues (this includes clarifying the links between the topics) 6. Conclude the interdisciplinary seminar (summarize, mention topics for further study if necessary, elicit feedback from the colleagues, allow time for online evaluation, and complete attendance cards).   ***… after*** the interdisciplinary seminar:   1. Follow-up of the interdisciplinary seminar (evaluation of feedback, revision of materials [e.g. presentation slides, group work, transitions between parts of the interdisciplinary seminar], joint reflection on one’s own priority setting and teaching performance).   **Task limitations:**  The performance of this teaching activity does not apply to content that does not belong to one’s own subject area. |
| **Teacher competencies domains:**  (X) information provider and coach, (X) facilitator and mentor, ( ) curriculum developer and implementer, ( ) assessor and diagnostician, (X) role model, ( ) manager and leader, ( ) scholar and researcher, (X) professional. |
| **Knowledge:**  Content knowledge in the topic of the interdisciplinary seminar; embedding of the topic in the sequence and structure of the Modular Curriculum of Medicine; expected prior knowledge of the students in the topic of the interdisciplinary seminar; structuring of the interdisciplinary seminar in terms of time and content; basic scientific concepts for the learning process (and for forgetting); principles of adult learning, student-centered and self-directed learning; importance and dimensions of learning objectives; alignment of teaching, learning and assessment based on learning objectives; concepts of effective teaching/learning and learning/learner diagnostics.  **Skills:**  Design of the interdisciplinary seminar according to the concept of teaching quality (main dimensions: transfer of knowledge and support of understanding, motivation and creation of a supportive learning environment; management of interactions in the learning group); learning/learner diagnostics at the beginning and during the interdisciplinary seminar; demonstration of relevance for the medical profession and adaptation of the design accordingly; assessment of the quality of the seminar (evaluation and examination results) and its continuous development; use of the Charité's learning management system (e.g. Blackboard, LLP).  **Attitudes:**  Enthusiasm for the subject and profession; awareness of one’s own (implicit and explicit) role model function; consideration of the diversity of students (e.g. differences in previous knowledge and learning speed); use of language appropriate to the students’ learning level; professional approach and respectful communication with the students. |

| **EPA 1.4: Teach a laboratory course** |
| --- |
| **Short description:**  The teacher prepares, teaches and follows up on a practical course for medical students in terms of content and didactics. |
| **Task specification:**  Performance of this teaching activity includes ...  ***… before* the practical course:**   1. Become informed about the content, possible practical activities, learning objectives, and embedding of the practical course (in the week and module theme and overall curriculum) and check that they are up to date. 2. Prepare for the sequence of the practical course (content arrangement, content of practical activities, time structure (pay attention to appropriate time for practice), use teaching methods and materials, be familiar with the experimental techniques (safety measures, and teaching materials) and take care of organizational matters.   ***… during* the practical course:**   1. Start the practical course (introduction, learning objectives, embedding in the module and in the curriculum, planned sequence). 2. Provide the students with the course content in an appropriate interactive way and support them in their learning process (explain the practical tasks and activities clearly to the students, guide and practice (e.g. microscopy, experiments, medical examinations), summarize, make connections, check understanding, observe the performance, convey scientific methods), give feedback to stimulate learning, allow and answer questions, use didactic aids in a targeted way) 3. Motivate the students and create a stimulating learning environment (create a good environment, encourage self-activity, clarify relevance, encourage, and strengthen self-efficacy). 4. Manage interactions in the student group when necessary for the learning process (assign roles and tasks manage communication, manage time, manage conflict effectively) and facilitate interaction between students and teachers (ask questions into the learning group, encourage students to ask questions). 5. Conclude the practical course (elicit feedback from students, allocate time for online evaluation, complete attendance cards).   ***… after* the practical course:**   1. Follow up on the practical course (evaluate feedback, revise the materials [e.g. practical course script, practical course models, reflect on one’s own priority setting and teaching performance).   **Task limitations:**  The performance of this teaching activity does not apply to content that does not belong to one’s own subject area. |
| **Teacher competencies domains:**  (X) information provider and coach, (X) facilitator and mentor, ( ) curriculum developer and implementer, ( ) assessor and diagnostician, (X) role model, ( ) manager and leader, ( ) scholar and researcher, (X) professional. |
| **Knowledge:**  Content knowledge in the topic of the practical course; knowledge of scientific inquiry methods; embedding of the topic in the sequence and structure of the Modular Curriculum of Medicine; expected prior knowledge of the students in the topic; structuring of the practical course in terms of time and content; basic scientific concepts for the learning process (and for forgetting); principles of adult learning, student-centered and self-directed learning; meaning and dimensions of learning objectives; alignment of teaching, learning and assessment based on learning objectives; concepts of effective teaching/learning and learning/learner diagnostics, formative assessment.  **Skills:**  Design of the practical course according to the concept of teaching quality (main dimensions: transfer of knowledge and support of understanding, motivation and creation of a supportive learning environment; management of interactions in the learning group); learning/learner diagnostics at the beginning of and during the practical course; demonstration of relevance for the medical profession and adaptation of the design accordingly; demonstration of technical expertise for the practical content of the course; assessment of the quality of the practical course (evaluation and examination results) and its continuous development; use of the Charité's learning management system (e.g. Blackboard, LLP).  **Attitudes:**  Enthusiasm for the subject and profession; awareness of one’s own (implicit and explicit) role model function; consideration of the diversity of students (e.g. differences in previous knowledge and learning speed); use of language appropriate to the students’ learning level; professional approach and respectful communication with the students. |

| **EPA 1.5: Teach a bedside teaching course** |
| --- |
| **Short description:**  The teacher prepares, teaches and follows up a bedside teaching course for medical students in terms of content and didactics. |
| **Task specification:**  Performance of this teaching activity is based on the bedside teaching schedule at the Charité (6 steps) and includes ...  ***… before*** the bedside teaching course:  (**Step I**: Preparation)   1. Become informed about the content, learning objectives and embedding of the bedside teaching course (in the week and module topic and overall curriculum) and check that they are up to date. 2. Prepare for the sequence of the bedside teaching course process (identify topic, select suitable patients, prepare and agree on terms with patients, agree on terms with the team, prepare materials and the room).   ***… during*** the bedside teaching course:   1. Start the bedside teaching course (mention the subject of the session, discuss the embedding in the module, describe the planned sequence, discuss specific learning objectives/needs, assign roles and tasks to students, address topics/behaviors to avoid in patient contact).   (**Step II:** Briefing)   1. Guide the students through further steps of bedside teaching (ensure compliance with the planned bedside teaching steps, provide assistance if necessary, adjust supervision level):   **Step III:** Contacting the patient; **Step IV:** Preparing the patient presentation  **Step V:** Presenting the patient; **Step VI:** Demonstrating findings and guiding a final discussion   1. Provide the students with the course content and practical skills to in an appropriate way and support them in their learning process (present in a clear and structured way, explain clearly to students, summarize and repeat, make links, check understanding, give feedback to stimulate learning, use didactic aids appropriately). 2. Ensure patient safety and well-being. 3. Motivate the students and create a stimulating learning environment (arouse interest, clarify relevance, provide encouragement, formulate appropriate task requirements and build self-efficacy). 4. Manage interactions between the students and the patients (manage communication and time, deal effectively with disruptins), give feedback to the students on their interaction with the patients. 5. Conclude the bedside teaching course (obtain feedback from students, allocate time for online evaluation, complete attendance cards).   In later semesters, some of the teacher’s tasks (Step 3-5) should be taken over by the students.  ***… after*** the bedside teaching course:   1. Follow up of the bedside teaching course (evaluate feedback, revise the materials [e.g. selection of patients), obtain feedback from the patients, reflect on one’s own priority seeting and teaching performance).   **Task limitations:**  The performance of this teaching activity does not apply to content that does not belong to one’s own subject area. |
| **Teacher competencies domains:**  (X) information provider and coach, (X) facilitator and mentor, ( ) curriculum developer and implementer, (X) assessor and diagnostician, (X) role model, ( ) manager and leader, ( ) scholar and researcher, (X) professional |
| **Knowledge:**  Content knowledge in the topic of the bedside teaching course; embedding of the topic in the sequence and structure of the Modular Curriculum of Medicine; expected prior knowledge and clinical skill level of the students in the topic; structuring of the bedside teaching course in terms of time and content, principles of patient suitability and selection at the Charité; steps and methods of clinical reasoning and differential diagnosis; basic scientific concepts for the learning process (and for forgetting); principles of adult learning, student-centered and self-directed learning; importance and dimensions of learning objectives; alignment of teaching, learning and assessment based on learning objectives; concepts of effective teaching/learning and learning/learner diagnostics, formative assessment, principles of effective feedback, importance of the teacher as a role model in clinical teaching settings.  **Skills:**  Design of the course according to the concept of bedside teaching at the Charité; learning/learner diagnostics at the beginning of and during the bedside teaching course; demonstration of relevance for the medical profession and adaptation of the design accordingly; teaching of practical skills, observation and provision of feedback; assessment of the quality of the bedside teaching course (evaluation and examination results) and continuous development of it; use of the Charité's learning management system (e.g. Blackboard, LLP).  **Attitudes:**  Enthusiasm for the subject and the profession; awareness of one’s own (implicit and explicit) role model function; treatment of patients with respect; use of language that is understandable to patients; compliance with hygiene regulations; consideration and emphasis of privacy and the confidentiality of patient data; consideration of the diversity of students (e.g. differences in previous knowledge and speed of learning); use of language that is appropriate to the students’ learning stage; professional interaction and respectful communication with the students. |

| **EPA 1.6: Teach a communication, interaction and teamwork course** |
| --- |
| **Short description:**  The teacher prepares, teaches and follows up a communication, interaction and teamwork (CIT) course for medical students with and without the use of simulated patients in terms of content and didactics. |
| **Task specification:**  Performance of this teaching activity includes ...  ***… before* the CIT session:**   1. Become informed about the content and the learning objectives to be taught and the embedding of the session (in the week and module theme and overall curriculum), in particular in terms of the longitudinal CIT curriculum and simulated patient cases. 2. Prepare for the sequence of the session (based on the teacher’s handbook, content arrangement, time structure, teaching methods and materials, organizational matters, especially the time and agreement on content with simulated patient).   ***… during* the CIT session:**   1. Start the CIT session (introduction, learning objectives, embedding in the CIT longitudinal curriculum, and planned sequence). 2. Guide the students through the session according to the CIT teacher’s handbook and support them in their learning process (work out the content together, check understanding, give feedback to stimulate learning. 3. Prepare and revise the simulated patient discussions according to the given sequence (the discussion starts and ends in the room; once the discussion starts, the teacher becomes an observer and does not teach) ask for and give constructive feedback, deal professionally with simulated patients (in the sense of role modeling). 4. Motivate the students and create a good and safe learning environment (ensure confidentiality, arouse interest, avoid monotony, encourage self-activity, clarify relevance, encourage, formulate appropriate tasks). 5. Manage interactions in the group (manage communication and time, deal effectively with disruptions). 6. Close the CIT session (elicit feedback from the students, allocate time for online evaluation, complete attendance cards) and provide future prospects.   ***… after* the CIT session:**   1. Follow up on the CIT session (evaluate feedback, revise the materials [e.g. role play], reflect on one’s own priority setting and teaching performance).   **Task limitations:**  The performance of this teaching activity does not apply to content that does not belong to one’s own subject area. |
| **Teacher competencies domains:**  (X) information provider and coach, (X) facilitator and mentor, ( ) curriculum developer and implementer, ( ) assessor and diagnostician, (X) role model, ( ) manager and leader, ( ) scholar and researcher, (X) professional |
| **Knowledge:**  Knowledge of communication models; embedding of the topic in the sequence and structure of the Modular Curriculum of Medicine; expected prior knowledge and level of communication skills of students in the corresponding topic; time and content structuring of the schedule for CIT courses at the Charité; basic scientific concepts of learning (and for forgetting); principles of adult learning, student-centered and self-directed learning; meaning and dimensions of learning objectives; alignment of teaching, learning and assessment based on learning objectives; concepts of effective teaching/learning and learning/learner diagnostics; concepts of effective feedback; didactic knowledge for conducting role plays.  **Skills:**  Design of the session according to the CIT concept at the Charité; learning/learner diagnostics at the beginning of and during the CIT session; demonstration of relevance to the medical profession and adaptation of the instructional design accordingly; imparting of practical skills, observation and feedback; assessment of the quality of the CIT session (evaluation and examination results) and continuous development of it; respectful interaction with simulated patients; use of the Charité's learning management system (e.g. Blackboard, LLP).  **Attitudes:**  Enthusiasm for the subject and profession; awareness of one’s own (implicit and explicit) role model function; consideration of the diversity of students (e.g. differences in previous knowledge, learning speed and cultural background); use of language appropriate to the students’ learning level; professional approach and respectful communication with the students. |

| **EPA 1.7: Facilitate a problem-based learning group** |
| --- |
| **Short description:**  The teacher prepares and facilitates a problem-based learning (PBL) group according to the Berlin PBL concept in medical studies. |
| **Task specification:**  Performance of this teaching activity includes ...  ***… before*** the PBL session:   1. Familiarize oneself with the PBL case and become knowledgeable about the embedding of the session (week and module theme, overall curriculum).   ***… during*** the PBL session:   1. Start the PBL session (distribute the vignettes, explain the planned sequence, have the case read out). 2. Support the learners in their learning process (provide background information on the case if necessary, give methodological help, ensure that the results are secured and presented in a meaningful way by the learners, ensure that the given PBL steps are followed and guide the PBL steps:   Step 1: Clarify understanding  Step 2: Agree on the topic  Step 3: Activate prior knowledge Day 1  Step 4: Discuss previous knowledge and ideas  Step 5: Formulate learning goals  Step 6: Reflection  Step 7: Collect and discuss the learning content developed  Step 8: Reflect on the learning process Day 2   1. Motivate students and create a good learning environment (stimulate the PBL process and active discussions by providing information, observe and analyze teamwork and the learning process, encourage self-activity, ensure that learners formulate appropriate learning objectives, encourage students to reflect and give feedback). 2. Manage interactions in the PBL group (take the responsibility for guiding and timing the given steps, promote constructive exchanges in the group, ensure that learners explain clearly, involve all students, deal effectively with disruptions).   In later semesters, some of the teacher’s tasks (tasks 3-5) should be taken over by the students.  ***… after*** the PBL session:   1. Follow up on the PBL session (evaluate feedback and own teaching performance). 2. Communicate the learning objectives formulated by the PBL group to the PBL quality management team.   **Task limitations:**  The performance of this teaching activity does not apply to content that does not belong to the medical fieldt. |
| **Teacher competencies domains:**  ( ) information provider and coach, (X) facilitator and mentor, ( ) curriculum developer and implementer, ( ) assessor and diagnostician, (X) role model, (X) manager and leader, ( ) scholar and researcher, (X) professional |
| **Knowledge:**  Embedding of the PBL topic in the design and structure of the Modular Curriculum of Medicine; expected prior knowledge of the students in the corresponding topic; time and content structuring of the schedule of PBL sessions at the Charité; principles of adult learning, student-centered and self-directed learning; concepts of effective teaching/learning and learning/learner diagnostics, concepts of effective feedback.  **Skills:**  Facilitation of the session according to the concept of PBL sessions at the Charité; concepts of group moderation and processes of group dynamics; observation and feedback; assessment of the quality of the PBL session (evaluation and examination results) and its continuous development; use of the Charité's learning management systems (e.g. Blackboard, LLP).  **Attitudes:**  Enthusiasm for the profession and the PBL method; awareness of one’s own (implicit and explicit) role model function; consideration of the diversity of students (e.g. differences in previous knowledge and learning speed); use of language appropriate to the students’ learning level; professional approach and respectful communication with the students. |

| **EPA 1.8: Teach a clinical skills course** |
| --- |
| **Short description:**  The teacher prepares, delivers and follows up on a clinical skills course for medical students in terms of content and didactics. |
| **Task specification:**  Performance of this teaching activity includes ...  ***… before*** the clinical skills course:   1. Become informed about the content and learning objectives of the examination course (week and module topics, overall curriculum). 2. Prepare for the sequence of the skills course (topic, preparation and agreement with patients, arrangement with the care team, preparation of the materials and room).   ***… during*** the clinical skills course:   1. Start the examination course (present an introduction, identify the topic of the session, discuss the planned sequence, determine roles and tasks for the students). 2. Provide the students with the educational content and practical skills in an appropriate manner and support their learning process (if necessary practice practical skills among the students, demonstrate with patients, supervise the skills performed by the students). 3. Ensure patient safety and well-being. 4. Motivate the students and create a good learning environment (arouse interest, avoid monotony, encourage self-activity, clarify relevance, encourage, and strengthen self-efficacy, formulate appropriate tasks). 5. Manage interactions within the student group and interactions between the student group and the patient (manage communication and time, deal effectively with disruptions). 6. Conclude the examination course (obtain feedback from students, allow time for online evaluation, complete attendance cards).   ***… after* the** clinical skills course:   1. Follow up on the examination course (evaluate feedback, revise the materials, obtain feedback from patients, reflect on own priority setting and teaching performance).   **Task limitations:**  The performance of this teaching activity does not apply to content that does not belong to one’s own subject area. |
| **Teacher competencies domains:**  (X) information provider and coach, (X) facilitator and mentor, ( ) curriculum developer and implementer, ( ) assessor and diagnostician, (X) role model, ( ) manager and leader, ( ) scholar and researcher, (X) professional |
| **Knowledge:**  Content knowledge in the subject of the skills course; embedding of the topic in the sequence and structure of the Modular Curriculum of Medicine; expected prior knowledge and clinical skill level of the students in the subject; structuring of the skills course in terms of time and content according to the schedule of skills courses at the Charité; principles of patient suitability and selection at the Charité; steps and methods of clinical reasoning and differential diagnosis; basic scientific concepts for the learning process (and for forgetting); principles of adult learning, student-centered and self-directed learning; importance and dimensions of learning objectives; alignment of teaching, learning and assessment based on learning objectives; concepts of effective teaching/learning and learning/learner diagnostics; principles of effective feedback.  **Skills:**  Conducting the course according to the concept of skills teaching at the Charité; learning/learner diagnostics at the beginning of and during the skills course; demonstration of relevance for the medical profession and adaptation of the design accordingly; teaching of practical skills; observation and feedback; elicitation of feedback from patients; assessment of the quality of the skills course (evaluation and examination results) and continuous development of it; use of the Charité's learning management systems (e.g. Blackboard, LLP).  **Attitudes:**  Enthusiasm for the subject and the profession; awareness of one’s own (implicit and explicit) role model function; respectful treatment of patients; use of language understandable to patients; compliance with hygiene regulations; consideration and emphasis of privacy and the confidentiality of patient data; consideration of the diversity of students (e.g. differences in previous knowledge and speed of learning); use of language appropriate to the students’ learning stage; professional interaction and respectful communication with the students. |

| **EPA 1.9: Teach an emergency simulation course** |
| --- |
| **Short description:**  The teacher prepares, conducts and follows up together on an emergency simulation course for medical students in terms of content and didactics. |
| **Task specification:**  Performance of this teaching activity includes ...  ***… before*** the emergency simulation course:   1. Become informed about the content and learning objectives of the course (in particular regarding other specialized and interdisciplinary simulations, the week and module themes, overall curriculum). 2. Prepare for the sequence of the emergency simulation course (content and timing, functionality of the equipment, organizational issues).   ***… during*** the emergency simulation course:   1. Start the emergency simulation course (introduction, learning objectives, embedding in the module, the curriculum and the emergency weeks, explanation of the planned sequence, explanation of the sequence of the individual scenarios in the first simulation session, familiarization with the available material (e.g., simulators, briefing) 2. Provide the students with the course content in an appropriate way and support their learning process (lead the scenarios in a clear and structured way, conduct an appreciative debriefing, summarize the most important aspects of the scenario, identify further learning needs, lead to the next scenario). 3. Motivate the students and create a good learning environment (formulate appropriate tasks, arouse interest, avoid monotony, encourage self-activity in terms of competence to act in emergency situations, encourage self-directed learning, clarify relevance, provide constructive feedback and fair debriefing). 4. Manage interactions in the student group (manage communication, include the role of nontechnical skills, time, deal effectively with disruption). 5. Conclude the emergency simulation session (elicit feedback from students, allow time for online evaluation, complete attendance cards).   ***… after*** the emergency simulation course:   1. Follow up on the emergency simulation course (evaluate feedback, revise the materials (e.g. presentations), reflect on one’s own priority setting and teaching performance).   **Task limitations:**  The performance of this teaching activity does not apply to content that does not belong to one’s own subject area. |
| **Teacher competencies domains:**  (X) information provider and coach, (X) facilitator and mentor, ( ) curriculum developer and implementer, ( ) assessor and diagnostician, (X) role model, ( ) manager and leader, ( ) scholar and researcher, (X) professional. |
| **Knowledge:**  Content knowledge in emergency medicine; embedding of the topic in the sequence and structure of the Modular Curriculum of Medicine; expected prior knowledge and clinical skill level of the students in the topic; structuring of the emergency simulation course in terms of time and content according to the schedule of specialized simulation courses at the Charité; steps and methods of clinical reasoning and differential diagnosis; basic scientific concepts for the learning process (and for forgetting); principles of adult learning, student-centered and self-directed learning; importance and dimensions of learning objectives; alignment of teaching, learning and assessment based on learning objectives; concepts of effective teaching/learning and learning/learner diagnostics; principles of effective feedback.  **Skills:**  Conducting of the course in accordance with the Charité concept of specialized simulation; learning/learner diagnostics at the beginning of and during the specialized simulation; demonstration of relevance to the medical profession and adaptation of the design accordingly; teaching of practical skills, observation and feedback; assessment of the quality of the specialized simulation course (evaluation and examination results) and continuous development of it; use of the Charité's learning management systems (e.g. Blackboard, LLP).  **Attitudes:**  Enthusiasm for the subject and the profession; awareness of one’s own (implicit and explicit) role model function; compliance with hygiene regulations; consideration of the diversity of students (e.g. differences in previous knowledge and learning speed); use of language appropriate to the students’ learning stage; professional interaction and respectful communication with the students. |

| **EPA 1.10: Teach an interdisciplinary emergency simulation course** |
| --- |
| **Short description:**  The teacher prepares, conducts and follows up together with a co-teacher on an emergency simulation course for medical students in terms of content and didactics. |
| **Task specification:**  Performance of this teaching activity includes ...  ***… before*** the interdisciplinary emergency simulation course:   1. Become informed about the content, learning objectives and embedding of the session (especially in relation to other specialized and interdisciplinary simulations, week and module theme, overall curriculum). 2. Prepare for the sequence of the simulation in collaboration with the co-teacher (content and timing, functionality of the equipment, organizational matters).   ***… during*** the interdisciplinary emergency simulation course:   1. Start the interdisciplinary simulation session together with the co-teacher (introduction, learning objectives, embedding in the module and curriculum and in the emergency weeks, planned sequence), explain the general sequence of the individual scenarios in the first simulation session, familiarize the students with the available material (e.g., simulators), briefing). 2. Provide the students with the course content in an appropriate manner and support them in their learning process (lead the scenarios in a clear and structured manner, conduct an appreciative debriefing, summarize the most important aspects of the scenario, identify further learning needs, lead to the next scenario). 3. Use the different professional backgrounds of the teachers to discuss the different therapies for different emergencies. 4. Motivate the students and create a good learning environment (formulate appropriate tasks, arouse interest, avoid monotony, encourage self-activity in terms of competence to act in emergency situations, encourage self-directed learning, clarify relevance, give constructive feedback and fair debriefing). 5. Manage interactions in the student group (manage communication, include the role of nontechnical skills, and time, deal effectively with disruption). 6. Conclude the interdisciplinary emergency simulation session together with the co-teacher (obtain feedback from students, allocate time for online evaluation, complete attendance cards).   ***… after*** the interdisciplinary emergency simulation course:   1. Follow-up of the interdisciplinary emergency simulation course in collaboration with the co-teacher (evaluate feedback, revise the materials (e.g. presentations), reflect on one’s own priority settings and teaching performance).   **Task limitations:**  The performance of this teaching activity does not apply to content that does not belong to one’s own subject area. |
| **Teacher competencies domains:**  (X) information provider and coach, (X) facilitator and mentor, ( ) curriculum developer and implementer, ( ) assessor and diagnostician, (X) role model, ( ) manager and leader, ( ) scholar and researcher, (X) professional |
| **Knowledge:**  Content knowledge in the subject of emergency medicine; embedding of the subject of emergency in the sequence and structure of the Modular Curriculum of Medicine; expected level of prior knowledge and clinical skills level of the students in the subject; structuring of the emergency simulation course in terms of time and content according to the schedule of interdisciplinary simulation courses at the Charité; steps and methods of clinical reasoning and differential diagnosis; basic scientific concepts for the learning process (and for forgetting); principles of adult learning, student-centered and self-directed learning; importance and dimensions of learning objectives; alignment of teaching, learning and assessment based on learning objectives; concepts of effective teaching/learning and learning/learner diagnostics, principles of effective feedback.  **Skills:**  Conducting the course according to the concept of the interdisciplinary simulation at the Charité; learning/learner diagnostics at the beginning of and during the interdisciplinary simulation; demonstration of relevance for the medical profession and adaptation of the design accordingly; teaching of practical skills, observation and feedback; assessment of the quality of the interdisciplinary simulation course (evaluation and examination results) and its continuous development; use of the Charité's learning management systems (e.g. Blackboard, LLP).  **Attitudes:**  Enthusiasm for the subject and the profession; awareness of one’s own (implicit and explicit) role model function; consideration of the diversity of students (e.g. differences in previous knowledge and speed of learning); use of language appropriate to the students’ learning stage; professional interaction and respectful communication with the students. |

| **EPA 2.1: Supervise a medical student in an early clerkship placements** |
| --- |
| **Short description:**  The teacher introduces a medical student to the medical workplace in the respective placement and guides and supervises the performance of his/ her medical tasks. |
| **Task specification:** |
| Performance of this activity includes ...  ***… before*** the start of the early clerkship placement:   1. Welcomes the student and introduces him/ her to the ward or outpatient clinic (health care team, overview of the range of complaints, diseases, procedures, therapies, learning opportunities). 2. Communicates what the medical student is allowed to do by him-/ herself (with supervisor follow-up check) or together with a physician or are not allowed to perform (considering previous clinical and practical experience and/or the EPAs already performed). 3. Describes the expectations of what is to be achieved in the clinical placement (tasks and levels of autonomy/supervision) and to identify the gaps compared to the intended outcomes at the end of the clinical placement.   ***… during*** the early clerkship placement*:*   1. Motivates the student and create a supportive learning environment (arouse interest, clarify relevance, encourage, formulate appropriate task requirements, encourage self-activity). 2. Assigns appropriate tasks of increasing difficulty to the student. 3. Guides the student through unfamiliar tasks (instruct, co-perform, observe, and provide feedback). 4. Supervises tasks carried out autonomously by the student (checking work results). 5. Provides the student with a place to work and study. 6. Actively integrates the student into the team (assigns roles and tasks, manages communication and time, deals, effectively with disruption).   ***… after*** the early clerkship placement:   1. Discusses the learning outcomes (tasks and level of autonomy/supervision) achieved by the student during the clinical placement and addresses any gaps.   **Task limitations:**  The performance of this teaching activity does not apply to content that does not belong to one’s own subject. |
| **Teacher competencies domains**  ( ) information provider and coach, (X) facilitator and mentor, ( ) curriculum developer and implementer, (X) assessor and diagnostician, (X) role model, ( ) manager and leader, ( ) scholar and researcher, (X) professional |
| **Knowledge:**  Content knowledge of the relevant clinical area (range of complaints, diseases, procedures, therapies); expected level of prior knowledge and clinical skills level of the student; principles of effective teaching, learning and assessment in the clinical workplace; concept of clinical placement and 1:1 teaching; structuring of learning and participation of the student in real patient care settings; use of Entrustable Professional Activities (EPAs) in a clinical context; objectives for the clinical placement (range of tasks to be performed and level of autonomy/ supervision achieved); steps and teaching of clinical reasoning in differential diagnosis and treatment decisions; principles of effective feedback; the importance of being implicit and explicit role modeling  **Skills:**  Introduction of the student to the workplace and the team; specification of the range of tasks and the level of autonomy/ supervision; identification of learning opportunities during the clinical placement in the relevant specialty; setting of objectives to be achieved by the end of the clinical placement; provision of practical skills; observation and feedback; support of the student’s professional development; final discussion with a reflection on what has been achieved and what is being aspired to in the future.  **Attitudes:**  Enthusiasm for the subject and profession; awareness of being an (implicit and explicit) role model; respectful treatment of patients and colleagues in the (interprofessional) health care team; compliance with hygiene regulations; respect and emphasis on privacy and confidentiality of patient information, attention to the diversity of students (e.g. differences in prior knowledge and learning speed); professional interactions and respectful communication with trainees; role as a teacher, coach and mentor for the professional development of the student trainees. |

| **2.2: Supervise a medical students in a short-block clinical placements** |
| --- |
| **Short description:**  The teacher introduces a medical student to the workplace and guides and supervises the performance of his/ her medical tasks. |
| **Task specification:** |
| Performance of this activity includes ...  ***… before*** the start of the short-block clinical placement:   1. Welcomes the medical student and introduce him / her to the ward or outpatient clinic (health care team, overview of the range of complaints, diseases, procedures, therapies, learning opportunities). 2. Communicates what the medical student is allowed to do by him-/ herself (with supervisor follow-up check) or together with a physician or are not allowed to perform (considering previous clinical and practical experience and/or the EPAs already performed). 3. Describes the expectations of what is to be achieved in this placement (tasks and levels of autonomy/ supervision) and to identify the gaps compared to the intended outcomes at the end of the placement.   ***… during*** the short-block clinical placement*:*   1. Motivates the medical student and create a supportive learning environment (arouse interest, clarify relevance, encourage, formulate appropriate task requirements, encourage self-activity). 2. Provides the medical student with appropriate tasks of increasing difficulty. 3. Guides the medical student through unfamiliar tasks (instruct, co-perform, observe and provide feedback). 4. Supervises tasks performed autonomously by the medical student (check work results). 5. Provides the medical student with a place to work and study. 6. Actively integrates the medical student into the team (assigns roles and tasks, manages communication and time, deals effectively with disruption).   ***… after*** the short-block clinical placement:   1. Discusses the learning outcomes achieved by the medical student during the placement (tasks and level of autonomy/ supervision) and addresses any gaps.   **Task limitations:**  The performance of this teaching activity does not apply to content that does not belong to one’s own subject. |
| **Teacher competencies domains:**  ( ) information provider and coach, (X) facilitator and mentor, ( ) curriculum developer and implementer, ( ) assessor and diagnostician, (X) role model, ( ) manager and leader, ( ) scholar and researcher, (X) professional |
| **Knowledge:**  Content knowledge in the relevant clinical area (range of complaints, diseases, procedures, therapies); expected level of prior knowledge and clinical skills level of medical students in the short-block clinical placements; principles of effective teaching, learning and assessment in the clinical workplace; concept of clinical teaching and 1:1 teaching; structuring of learning and participation of medical students in real patient care settings, using Entrustable Professional Activities (EPAs) in a clinical context; objectives for the short-block clinical placement (range of tasks to be performed and level of autonomy/ supervision achieved); steps and teaching of clinical reasoning in differential diagnosis and treatment decisions; principles of effective feedback; the importance of being implicit and explicit role modeling.  **Skills:**  Introduction of the medical student to the workplace and the team; explicit specification of the range of tasks and the level of autonomy/ supervision; identification of learning opportunities during the placement in the relevant specialty area; setting of objectives to be achieved by the end of the placement; teaching of practical skills; observation and feedback; support of the student in their professional development; final discussion with a reflection on what has been achieved and what is being aspired to in the future.  **Attitudes:**  Enthusiasm for the subject and the profession; awareness of being an (implicit and explicit) role model; respectful treatment of patients and colleagues in the (interprofessional) health care team, compliance with hygiene regulations; respect and emphasis on privacy and confidentiality of patient information; attention to the diversity of students (e.g. differences in prior knowledge and learning speed); professional interaction and respectful communication with students in the block internship placement; roles as a teacher, coach and mentor for the professional development of the students. |

| **EPA 2.3: Supervise a medical student in the final clerkship year placements** |
| --- |
| **Short description:**  The teacher instructs and supervises a medical student in the final clerkship year placement and prepares him/ her for the start of postgraduate medical training. |
| **Task specification:**  Performance of this activity includes ...  ***… before*** the start of final clerkship year rotation:   1. Welcomes the student and introduce Him /her to the ward or outpatient clinic (health care team, overview of the range of complaints, diseases, procedures, therapies, learning opportunities). 2. Communicates what the medical student are allowed to do by him- / herself (with supervisor follow-up check) or together with a physician or are not allowed to perform (considering previous clinical and practical experience and/or the EPAs already performed). 3. Describes the expectations of what should be achieved in the corresponding rotation of the practical year (tasks and level of autonomy/supervision) and show the gaps compared to the intended outcomes (educational objectives of the Charité) at the end of the final clerkship year.   ***… during*** the final clerkship year rotation:   1. Motivates the student and create a supportive learning environment (arouse interest, clarify relevance, encourage, formulate appropriate task requirements, encourage self-activity). 2. Assigns tasks of increasing difficulty to the student. 3. Guides the student through unfamiliar activities (instruct, co-perform, observe and provide feedback). 4. Supervises tasks carried out autonomously by the student (check work results). 5. Provides the final clerkship student with a place to work and study. 6. Actively integrates the student into the team (assigns roles and tasks, manages communication and time, deals effectively with disruptions).   ***… after*** the final clerkship year rotation:   1. Discusses he outcomes of the corresponding/respective final clerkship year rotation (tasks and level of autonomy/ supervision) and addresses any gaps in relation to the start of postgraduate medical training. 2. Reflect on one’s own performance (e.g., teaching style, feedback, time management).   **Task limitations:**  The performance of this teaching activity does not apply to content that does not belong to one’s own subject, |
| **Teacher competencies domains:**  ( ) information provider and coach, (X) facilitator and mentor, ( ) curriculum developer and implementer, (X) assessor and diagnostician, (X) role model, ( ) manager and leader, ( ) scholar and researcher, (X) professional |
| **Knowledge:**  Content knowledge in the relevant area (range of complaints, diseases, procedures, therapies); expected prior knowledge and level of clinical skills of a final clerkship year student; principles of effective teaching, learning and assessment in the clinical workplace; concept of clinical education and 1:1 teaching; structuring of learning and participation of practical year students in real patient care settings; use of Entrustable Professional Activities (EPAs) in a clinical context; objectives for the practical year (range of tasks to be performed and level of autonomy/ supervision achieved); steps and teachings of clinical reasoning in differential diagnosis and treatment decisions; principles of effective feedback; the importance of being implicit and explicit role modeling.  **Skills:**  Introduction of clerkship year student to the workplace and the team; explicit specification of the range of tasks and the level of autonomy/ supervision; identification of learning opportunities during the clerkship year in the relevant specialty; setting of objectives to be achieved by the end of the practical year; teaching of practical skills; observation and feedback; support of the student in their professional development; final discussion with a reflection on what has been achieved and what is being aspired to in the future.  **Attitudes:**  Enthusiasm for the subject and profession; awareness of being an (implicit and explicit) role model; respectful treatment of patients and colleagues in the (interprofessional) health care team; compliance with hygiene regulations; respect for and emphasis on privacy and the confidentiality of patient information; consideration of the diversity of students (e.g. differences in prior knowledge and speed of learning); professional interaction and respectful communication with the students; roles as a teacher, coach and mentor for the professional development of the final clerkship year students. |
