# Supplementary material for: Defining a set of teaching EPAs tailored to an undergraduate medical curriculum using a modified Delphi approach
Source: BMC Med Educ. 2024 May 28;24:588. doi: 10.1186/s12909-024-05553-5 (PMC11134953; doi:10.1186/s12909-024-05553-5)
Supplement: Supplementary file 2 — Supplementary Material 2. [file 12909_2024_5553_MOESM2_ESM.docx]

**Appendix II - “Defining a set of teaching EPAs tailored to**

**an undergraduate medical curriculum using a modified Delphi approach.”**

**Operationalisation of supervision levels and EPAs per study phase in the Modular Curriculum of Medicine at the Charité - Berlin**

**Levels of supervision**

| I. | allowed to observe, but not to perform the task |
| --- | --- |
| II. | direct supervision |
| II.a. | performs the tasks co-activity with the supervisor |
| II.b. | performs the task while the supervisor observes |
| III. | indirect supervision |
| III.a. | performs the task autonomously, with all/many findings double-checked by the supervisor afterwards |
| III.b. | performs the task autonomously, with key findings double-checked by the supervisor afterwards |

**EPAs for early clerkship placements**

(generally 4-6 weeks in duration)

| EPA No. | EPA title | Supervision level to be reached |
| --- | --- | --- |
| 1 | Take a medical history, perform a physical exam in a stable patient and provide a structured summary of the findings | III.a. |
| 2 | Present a patient history | II.b. |
| 4 | Perform general medical procedures |  |
| 4.1 | Sample venous or capillary blood | III.a. |
| 4.2 | Insert a peripheral venous catheter | III.a. |
| 4.3 | Take a blood culture | II.b. |
| 4.4. | Take a smear | II.b. |
| 4.5. | Write an ECG | II.a. |
| 4.6 | Handle a central venous catheter | II.b. |
| 4.7 | Take a blood culture | II.b. |
| 4.8 | Take a smear | III.b. |
| 4.9 | Place an urinary catheter | II.a. |
| 4.10 | Place a nasogastric tube | II.b. |

**EPAs for short-block clinical placements - example for the internal medicine placement**

(one week in duration for each discipline)

| EPA No. | EPA title | Supervision level to be reached |
| --- | --- | --- |
| 1 | Take a medical history, perform a physical exam in a patient admitted to an Internal Medicine ward and provide a structured summary of the findings | III.a. |
| 2 | Present a patient history | III.a. |
| 3 | Write a discharge report | II.a. |
| 4 | Perform general medical procedures |  |
| 4.1 | Sample venous or capillary blood | III.b. |
| 4.2 | Insert a peripheral venous catheter | III.a. |
| 4.3 | Take a blood culture | III.a. |
| 4.4. | Take a smear | III.b. |
| 4.5. | Write an ECG | II.a. |

**EPAs for the final clerkship year placement– example for the internal medicine placement**

(generally 16 weeks in duration)

| EPA No. | EPA title | Supervision level to be reached |
| --- | --- | --- |
| 1 | Take a medical history, perform a physical exam and provide a structured summary of the findings | III.b. |
| 2 | Present a patient history | III.b. |
| 3 | Compile a diagnostic work plan and initiate its implementation | III.a. |
| 4 | Seek consent for medical tests and procedures | II.a. |
| 5 | Interpret test results and initiate further steps | III.b. |
| 6 | Compile a treatment plan and initiate its implementation | III.a. |
| 7 | Inform and advise a patient | II.a. |
| 8 | Give a patient handover | II.b. |
| 9 | Recognize an emergency situation and act upon it | III.a. |
| 10 | Discharge a patient | III.a. |
| 11 | Perform general medical procedures |  |
| 11.1 | Sample venous or capillary blood | III.b. |
| 11.2 | Insert a peripheral venous catheter | III.b. |
| 11.3 | Handle a central venous catheter | III.b. |
| 11.4 | Take a blood culture | III.b. |
| 11.5 | Take a smear | III.b. |
| 11.6 | Place an urinary catheter | II.b. |
| 11.7 | Give an intravenous or intramuscular injection | II.b. |
| 11.8 | Place a nasogastric tube | II.b. |
| 11.9 | Perform a bed-site test to determine the blood group | II.a. |
| 11.10 | Sample arterial blood | II.a. |
| 11.11 | Perform a pleural puncture or ascites puncture | II.a. |
